# NADPH Oxidase as a Therapeutic Target for Neuroprotection against Ischaemic Stroke: Future Perspectives

**DOI:** 10.3390/brainsci3020561

**Published:** 2013-04-22

**Authors:** Sarah K. McCann, Carli L. Roulston

**Affiliations:** 1 Stroke Injury and Repair Team, O’Brien Institute, St Vincent’s Hospital, 42 Fitzroy St, Fitzroy, Melbourne 3065, Australia; E-Mail: sarah.mccann@florey.edu.au; 2 Florey Neuroscience Institutes, Melbourne Brain Centre, 245 Burgundy St, Heidelberg 3084, Australia; 3 Department of Medicine, University of Melbourne, St Vincent’s Campus, Melbourne 3065, Australia

**Keywords:** brain injury, cerebrovascular event, reactive oxygen species, Nox, brain repair, drug development, inhibitors, ischaemic stroke

## Abstract

Oxidative stress caused by an excess of reactive oxygen species (ROS) is known to contribute to stroke injury, particularly during reperfusion, and antioxidants targeting this process have resulted in improved outcomes experimentally. Unfortunately these improvements have not been successfully translated to the clinical setting. Targeting the source of oxidative stress may provide a superior therapeutic approach. The NADPH oxidases are a family of enzymes dedicated solely to ROS production and pre-clinical animal studies targeting NADPH oxidases have shown promising results. However there are multiple factors that need to be considered for future drug development: There are several homologues of the catalytic subunit of NADPH oxidase. All have differing physiological roles and may contribute differentially to oxidative damage after stroke. Additionally, the role of ROS in brain repair is largely unexplored, which should be taken into consideration when developing drugs that inhibit specific NADPH oxidases after injury. This article focuses on the current knowledge regarding NADPH oxidase after stroke including *in vivo* genetic and inhibitor studies. The caution required when interpreting reports of positive outcomes after NADPH oxidase inhibition is also discussed, as effects on long term recovery are yet to be investigated and are likely to affect successful clinical translation.

## 1. Introduction

Ischaemic stroke is a highly complex disorder characterised by multiple mechanistic pathways which contribute to brain cell death, and subsequent functional deficits in sufferers. Therapeutic options for victims of stroke remain limited and predominantly involve measures to recanalise ischaemic regions. Efforts to provide direct neuroprotection to brain cells at risk of death have largely been unsuccessful. The failure of the majority of experimental work to translate into effective treatment modalities in humans with ischaemic stroke is well recognised and has been analysed in multiple reviews and meta-analyses [1,2,3,4,5,6]. The overlapping nature of several important injury mechanisms means that a combination of approaches that target specific stages of ischaemic injury evolution may be required to achieve positive outcomes. It has been recommended that the pathways contributing to the progression of cell death be more completely characterised, in order to allow specific therapeutic targets to be identified and validated [7,8]. 

While restoring circulation is an important component in preventing stroke-related disability, halting the underlying mechanisms of neuronal damage with neuroprotective agents may play a central role in salvaging neuronal tissue. Pharmacological strategies aimed at limiting the delayed phase of ischaemic damage are important in stroke therapy as most patients are not diagnosed or treated early enough to prevent initial damage. The limited timeframe for safe administration and multiple contraindications mean that, on average, fewer than 5% of patients are eligible for treatment with tissue plasminogen activator (tPA), which is the only therapeutic for ischaemic stroke approved for use in most countries. The goal of tPA is to restore blood flow to ischaemic neurons by clot lysis, resulting in reperfusion of the ischaemic region Those patients that do experience reperfusion, either spontaneously or via tPA treatment, may derive benefit from adjuvant or combination therapy to protect neurons against additional injury that may occur as a result of reperfusion, or to interfere with cell death signaling pathways that may have already been initiated. 

Oxidative stress has been widely implicated in the progression of brain injury following ischaemia, and based on pre-clinical data delayed treatment with antioxidant neuroprotective agents may have potential to significantly reduce the disabling burden of stroke. However, antioxidant compounds such as AstraZeneca’s nitrone drug, NXY-059, failed to progress to clinical success [9,10]. Perhaps in light of this it should be recognised that the majority of antioxidants target reactive oxygen species (ROS) only after they are formed. Given that ROS are highly reactive and can cause damage rapidly, therapeutic strategies that target the source of ROS production may offer better neuroprotection than classical antioxidants. Invading neutrophils and macrophages that undergo a respiratory burst during reperfusion [11,12], glial cells [13], cerebrovascular cells [14,15], and importantly, neurons themselves [16,17,18] are potential sources of oxidative stress following injury to the brain. Several candidate sources of excessive levels of ROS in these cells exist, including intracellular organelles (especially mitochondria), xanthine oxidase, cyclooxygenases, lipoxygenases, cytochrome P450, substrate-uncoupled nitric oxide synthase (NOS), and NADPH oxidase [19,20,21,22,23,24]. It is also apparent that there exists a dynamic relationship between these ROS-generating systems in relation to changes in intracellular biochemistry. However, *in vitro* evidence suggests that in neurons three distinct phases of ROS generation occur with specific temporal relationships to metabolic events taking place during oxygen-glucose deprivation (OGD) and re-oxygenation: The first and second superoxide bursts were identified as arising from the mitochondria and xanthine oxidase, respectively, during the OGD phase. The third and final phase of sustained ROS generation was seen only upon re-oxygenation, was calcium dependent, and blocked only by inhibitors of NADPH oxidase [25]. Given that NADPH oxidase reduces molecular oxygen to superoxide, the lack of oxygen and glucose during ischaemia limits this reaction. Mounting evidence supports a role for NADPH oxidases as the major source of oxidative stress during post-ischaemic reperfusion [26,27]. Indeed, NADPH oxidases are the only enzyme family dedicated solely to ROS generation. This article will therefore review the current knowledge regarding NADPH oxidase and its related isoforms, as well as current inhibitors of NADPH oxidases and their potential as neuroprotectants. Finally, discussion will cover the caution needed in interpreting the positive outcomes observed with inhibitors of NADPH oxidase in pre-clinical stroke models, given that their effects on subsequent repair mechanisms, as well as long term systemic effects are yet to be fully characterised. This will be highly important for advancing the development of therapeutics that target NADPH oxidase so that any beneficial outcomes observed are not merely transient, and outweigh any potential side effects associated with systemic delivery and long term recovery.

## 2. Oxidative Stress

Under pathophysiological conditions, ROS react irreversibly with several cellular constituents including proteins, phospholipids and nuclear DNA, causing lipid peroxidation, membrane damage, dysregulation of cellular processes and mutations of the genome [28]. In addition to direct oxidative damage, ROS also act as important cellular signaling molecules that regulate changes in gene expression during ischaemia and initiate inflammation, apoptosis and blood brain barrier (BBB) disruption. In contrast, ROS signaling can also lead to the initiation or fostering of protective and regenerative mechanisms after stroke such as angiogenesis [29]. The brain may be especially vulnerable to oxidative stress for a number of reasons: It is highly enriched with polyunsaturated fatty acids, which are susceptible to attack from free radicals. Parts of the brain contain high levels of iron, which can participate in increased ROS generation [30]. Despite accounting for only around 2% of body weight, a high level of metabolic activity means the brain uses approximately 20% of total oxygen consumption at rest, providing ample substrates for ROS production [31]. Finally, given these factors, the brain is relatively poorly endowed with some protective antioxidant enzymes, including superoxide dismutase, catalase and glutathione peroxidase, when compared to other tissues [32,33]. Endogenous antioxidant systems regulate the level of ROS available for biological reactions under physiological conditions and represent a defence system against excessive ROS under pathophysiological conditions. However during post-ischaemic reperfusion these systems can become perturbed and overwhelmed by excess ROS production, leading to oxidative damage. There are several antioxidant systems in the brain catering for different ROS species in various cellular compartments. These include superoxide dismutases (SODs), a class of enzyme that catalyses the dismutation of superoxide to hydrogen peroxide and oxygen. The SOD family includes three isoforms: SOD1/Cu/ZnSOD, found in the cytosol, SOD2/MnSOD in mitochondria and SOD3/ecSOD in the extracellular space, cerebrospinal fluid and cerebral vessels [34]. Catalase and glutathione peroxidase are also naturally occurring antioxidants, and work by reducing hydrogen peroxide to water and oxygen. Catalase is localised mainly in peroxisomes while glutathione peroxidase is present in the cytosol [19]. Other small molecule antioxidants including glutathione, ascorbic acid and α-tocopherol are also involved in the detoxification of free radicals in the brain [34].

Because of the transient nature of ROS and the technical difficulties inherent in accurately measuring levels in the brain, experimental strategies to gauge their role in ischaemic stroke have included antioxidant treatments and the use of genetically modified animal models. The importance of ROS production in different brain compartments in contributing to oxidative stress has been demonstrated by the protection afforded against ischaemic injury in transgenic mice with enhanced ROS scavenging ability due to genetic overexpression of the antioxidant enzymes SOD1, SOD2 or SOD3 [35,36,37,38,39]. Complementary findings are obtained when these SOD isoforms are knocked out, or genetically deleted, resulting in an increase in ischaemic brain damage [40,41,42]. The protective effect of the endogenous antioxidant glutathione peroxidase after ischaemic stroke has also been demonstrated in transgenic mice overexpressing the enzyme or knockout mice lacking enzyme expression [43,44]. Interestingly, when transgenic mice that overexpress SOD were crossed with glutathione peroxidase knockout mice, the resulting offspring did not exhibit the protection afforded SOD transgenic mice [45]. These results highlight the importance of downstream ROS such as hydrogen peroxide, in addition to superoxide, and the synergistic roles played by the endogenous antioxidant defences in protecting the brain against ischaemic stroke and related conditions [45,46]. In addition to these studies using genetic manipulation, studies describing the administration of exogenous free radical scavengers such as SOD1, catalase, ascorbic acid, and α-tocopherol showed reduced neuronal injury after cerebral ischaemia and reperfusion [47,48,49]. However, the role of these exogenous antioxidants in protecting against stroke damage in humans is still subject to controversy [50,51].

## 3. Antioxidants for Stroke

The ischaemic brain is particularly vulnerable to attack from free radicals, and oxidative stress is thought to play a major role in brain injury after ischaemic stroke, and especially after reperfusion [52]. Administration of therapeutic antioxidants has yielded promising pre-clinical data: numerous compounds including inhibitors of free radical production, free radical scavengers and antioxidants that increase free radical degradation have declared up to a 90% reduction in infarct volume in rodent stroke models [53]. However, for the most part, these results have failed to translate into clinically effective treatments. 

One of the most widely cited failures in antioxidant neuroprotection is the free radical scavenger, NXY-059, tested in the Stroke-Acute Ischaemic NXY Treatment (SAINT) clinical trials. NXY-059 was tested in animal ischaemic stroke studies designed specifically to adhere to the STAIR criteria for pre-clinical testing [54,55], and the SAINT I clinical trial reported positive outcomes. Despite this, the SAINT II trial failed to show any beneficial effect [10]. This failure cast a pall over animal models of stroke and their relevance and usefulness in developing therapies for stroke. However, while it was found that the reported efficacy of NXY-059 in animal models of stroke was confounded by study quality [5], an individual animal meta-analysis of treatment effects in pre-clinical studies revealed that it was in fact neuroprotective in the experimental setting, although bias may have resulted in efficacy being overstated [56]. Closer evaluation of the SAINT trials revealed that negative results could reflect weak antioxidant capacity, poor BBB penetration and lack of synergism with current thrombolysis treatment (tPA) [52]. In addition, an overly broad treatment window was used in the reported clinical trials, which outlasted the time window of efficacy observed in pre-clinical trials, perhaps leading to a lack of salvageable penumbral tissue in treated patients [52]. It is likely that other antioxidant therapies have suffered similar problems in the translation from experimental to clinical domains.

In contrast to the above, the antioxidant edaravone has been reported as beneficial in a clinical setting [57] and was licensed for use in Japan in 2001. Edaravone readily crosses the BBB, scavenges multiple radical species including superoxide and under experimental conditions inhibits important pathogenic mechanisms such as delayed neuronal death, microglia-induced neurotoxicity, long-term inflammation, and expression of vascular endothelial growth factor and MMP-9, when given within 12–36 h of stroke onset [58,59,60]. More extensive clinical trials are currently underway which may result in the approval of edaravone for use in additional countries [52]. Optimism also remains surrounding the use of flavonols for acute stroke treatment, which we have reviewed previously [61]. Extensive experimental and epidemiological evidence suggest that intake of food rich in antioxidant flavonoids (both flavonols and flavanols) is associated with improvement of endothelial function and better outcomes in the face of cardiovascular disease [62,63]. Several compounds including quercetin, fisetin and DiOHF (3′,4′-dihydroxyflavonol) have been investigated in our laboratory and by others for the treatment of ischaemic stroke, with promising results [64,65]. Numerous additional agents that display antioxidant properties and have been used in an ischaemic stroke setting, including other phenolic compounds and vitamins, are described in precedent reviews [66,67,68,69,70,71,72,73].

Although the future of antioxidant therapy still holds promise, ROS are highly reactive and scavenging toxic levels of free radicals after they have been generated is likely not the most effective way of limiting their impact on surrounding tissue. Therapies aimed at inhibiting free radical generation by targeting generators of ROS include xanthine oxidase inhibitors and COX-2 inhibitors [53]. However the contribution of COX-2 to significant ROS generation in the setting of ischaemic stroke has been questioned [74], and xanthine oxidase-derived ROS may primarily be important in the ischaemic phase of stroke [25]. In contrast, NADPH oxidases have been shown to be particularly important sources of ROS production during the reperfusion phase of stroke [25], and moreover, are the only sources of dedicated ROS production. Therefore, targeting NADPH oxidase may prove more effective in limiting oxidative stress effects after ischaemia with reperfusion. De-activation of free radicals by antioxidants, or preferably, inhibition of free radical generation, could potentially lead to a decrease in stroke complications as well as prolongation of the therapeutic time window in acute ischaemic stroke for recanalisation therapy.

## 4. NADPH Oxidase

Nicotinamide adenine dinucleotide phosphate (NADPH) oxidases are a class of enzyme whose primary function is the generation of ROS. Initially characterised in neutrophils, there are now seven oxidases recognised as part of the NADPH oxidase family, each distinctly expressed and regulated across a diverse range of cells and tissues. In addition to the originally established role of the phagocyte NADPH oxidase in host-defence, under normal conditions ROS generated by NADPH oxidase enzymes are now recognised as participating in a variety of fundamental physiological processes such as signal transduction [75,76,77].

Accumulating data also demonstrate that numerous pathophysiological processes are mediated by NADPH oxidase-derived ROS production, including hypertension, asthma, renal cancer, hepatitis, cataract, osteoporosis, and diabetes, reflecting the diversity of tissues and organs that rely on ROS for normal functioning [78]. In the central nervous system, NADPH oxidases are thought to contribute to pathophysiologies including Alzheimer’s disease, Parkinson’s disease, amyotrophic lateral sclerosis, multiple sclerosis, anxiety, epilepsy, and schizophrenia [77,78]. Oxidative stress is also recognised as a factor contributing to cell death following ischaemic stroke, and NADPH oxidase is increasing acknowledged as a major source of excess ROS under these conditions.

### 4.1. The Phagocyte NADPH Oxidase

In 1933 it was reported that a marked increase in leukocyte oxygen consumption occurred during the phagocytosis of bacteria [79]. Baehner and Nathan [80] recognised the clinical significance of this “respiratory burst”, linking it to the defect in phagocytes of patients suffering from chronic granulomatous disease (CGD) [81,82]. It was subsequently shown that superoxide, derived from NADPH oxidase, was generated by normal leukocytes but not those from CGD sufferers, under circumstances that suggested a role in bacterial killing [83,84,85]. Subsequently, the components of NADPH oxidase were elucidated over several decades [86].

**Figure 1:**
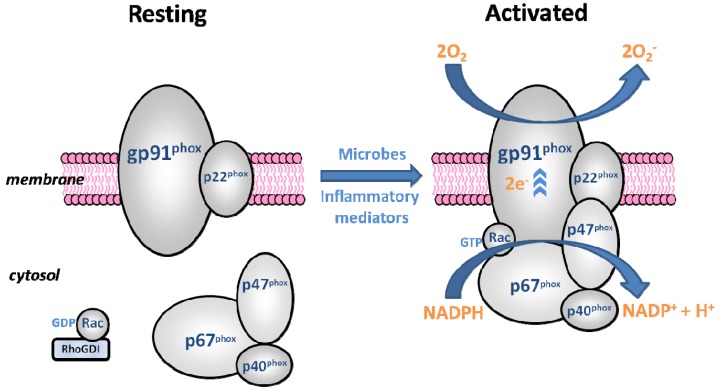
Diagrammatic representation of the resting and activated forms of the phagocytic NADPH oxidase. The catalytic subunit, gp91phox, along with p22phox makes up flavocytochrome b558, the membrane-associated component of the enzyme. The three phox proteins, p47, p67 and p40 form a cytosolic complex in the resting cell, and upon activation translocate to the membrane, docking with flavocytochrome b558. The small G-protein Rac, in its GDP-bound form, is stabilised by RhoGDI in the resting state and also translocates to the membrane upon activation. When assembled, the enzyme generates the ROS superoxide (O_2_^−^) by accepting electrons (e^−^) from cytoplasmic NADPH and donating them to molecular oxygen (O_2_).

The catalytic core of the phagocyte NADPH oxidase (phox) is gp91phox, a membrane integrated glycoprotein [87]. Gp91phox forms a heterodimer with the smaller, membrane-associated p22phox protein and together they make up the central component of NADPH oxidase; flavocytochrome b558 [88]. Four cytosolic components also belong to the complex: p47phox, p67phox, p40phox and the small G-protein Rac1 or Rac2 [87]. Regulation of the phagocytic system relies on spatial segregation of these essential components. The enzyme is dissociated in resting cells but rapidly assembled upon activation by exposure to microbes or inflammatory mediators [89] (Figure 1).

The phagocytic oxidase is agonist dependent, with no constitutive activity, and transfers electrons and consumes oxygen almost instantaneously when stimulated [89].The respiratory burst involves the direct reduction of molecular oxygen by one electron, resulting in the formation of the superoxide anion. NADPH oxidase catalyses this reaction, converting NADPH to NADP^+^ by liberating two electrons and one proton. The proton remains in the cytoplasm, while the two electrons are transported through the plasma/phagosomal membrane, binding to two oxygen molecules and forming two superoxide anions in the extracellular or intraphagosomal space [90] (Figure 1).

### 4.2. Nox2 Homologues

NADPH oxidase was originally thought to be unique to phagocytes. It was subsequently discovered that superoxide production occurred in non-phagocytic cells in an NADPH oxidase-like fashion in the absence of gp91phox [91,92]. However, it was not until the expansion of the human genome sequence databases in the 1990s that the NADPH oxidase family of ROS-generating oxidases was identified and characterised. Seven genes encoding gp91phox homologues have been identified in the human genome: the NADPH oxidases, Nox1-5 (where gp91phox is renamed Nox2) and the Dual oxidases, Duox1 and Duox2 (or Nox6 and Nox7). Nox1 was the first Nox2 homologue to be cloned and is most abundant in intestinal epithelium [93]. Nox3 was originally described in foetal kidney [94] and is now thought to be expressed almost exclusively in the inner ear [95]. Nox4 was originally described as a renal oxidase (Renox) because of its high expression levels in the kidney [96] but is now more recognised for its vascular expression [97]. Nox5 is found primarily in lymph node, spleen and testis and is more distantly related to Nox2 than the other Nox proteins [98]. The Nox5 gene is not present in the mouse or rat genome [29]. The Duox proteins were cloned from human and porcine thyroid glands and originally designated ThOX1 and ThOX2 [99,100]. They are highly expressed in the thyroid and airway epithelium [89], while Duox2 is also present in lung and gastrointestinal tract epithelium [78].The name Dual oxidase reflects the fact that these proteins possess a unique extracellular domain in addition to their Nox-like portions [101]. 

As with Nox2, the Nox1, Nox3 and Nox4 proteins also form heterodimers with p22phox, which is essential for their activity [102]. In contrast, Nox5, Duox1 and Duox2 are activated and regulated directly by calcium [103]. The organiser and activator functions carried out by p47phox and p67phox in Nox2 are either absent or carried out by alternative proteins or molecules in the other Nox enzymes, for example the p47phox and p67phox homologues, Noxo1 (Nox organiser protein 1) and Noxa1 (Nox activator protein 1) [89,104,105,106]. Nox4 does not require phosphorylation of regulatory subunits and exhibits high constitutive activity [29]. However it has been shown that a novel regulatory protein, Poldip2, enhances Nox4 enzymatic activity in vascular cells [107]. When activated, all of the Nox proteins generate superoxide anions by single electron reduction of molecular oxygen; however under certain conditions the Nox4 NADPH oxidase may produce high levels of hydrogen peroxide constitutively, without releasing quantifiable levels of superoxide [108]. The Duox proteins produce hydrogen peroxide directly, without detectable levels of superoxide [109]. As with Nox2, the Nox1 and Nox3 proteins are generally agonist dependent, producing superoxide when the enzyme is stimulated and assembled. However, Nox1 activity may be partially constitutive and partially activable due to the lack of an autoinhibitory region and phosphorylation sites on Noxo1 [110].

## 5. NADPH Oxidase-Derived ROS

**Figure 2:**
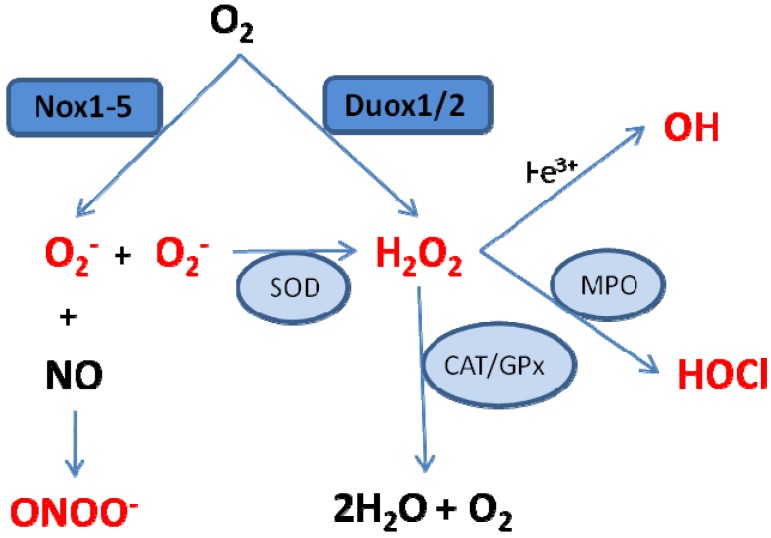
Simplified schematic illustrating the major pathways for the formation of ROS and reactive nitrogen species (RNS) originating from the Nox/Duox enzymes. Superoxide (O_2_^−^) is generated by the Nox enzymes and can be dismutated to hydrogen peroxide (H_2_O_2_), either spontaneously or catalysed by superoxide dismutase (SOD), or can react with nitric oxide (NO) to form peroxynitrite (ONOO^−^). H_2_O_2_, generated by the Duox enzymes or by dismutation of O_2_^−^ can be scavenged by the antioxidants catalase (CAT) or glutathione peroxidase (GPx) to form water (H_2_O) and oxygen (O_2_); be partially reduced to generate hydroxyl radical (OH) by the metal (Fe^3+^) catalysed Haber-Weiss and Fenton reactions; or react with chloride in a reaction catalysed by myeloperoxidase (MPO), resulting in formation of hypochlorous acid (HOCl).

The immediate products of the Nox/Duox enzymes are superoxide and hydrogen peroxide. These ROS have been implicated as cellular second messengers linked to signaling pathways and ion transport under physiological conditions [78]. Under pathological conditions, overproduction of ROS can disturb normal signaling pathways and exert damaging effects on target molecules. Moreover, in addition to these initial molecules, secondary reactive species can be formed through reaction with other nearby biomolecules (Figure 2). For example, dismutation of superoxide can occur, resulting in oxygen and hydrogen peroxide. This dismutation occurs spontaneously and is faster at lower pH levels, or can be catalysed enzymatically by SODs [90]. Superoxide also reacts rapidly with nitric oxide, resulting in the reactive nitrogen species (RNS) peroxynitrite, a highly potent oxidant. This process is highly regulated by the diffusion rate of both radicals and, in addition to the damaging effects of peroxynitrite, depletes nitric oxide levels available for alternative actions, for example vasodilation [78]. Hydrogen peroxide may be completely reduced to water and oxygen, a reaction catalysed by the antioxidant enzymes catalase and glutathione peroxidase, or partially reduced to hydroxyl anion, the most powerful oxidising agent identified in biological systems. The generation of hydroxyl anion is catalysed by free transition metal ions (e.g., Fe^3+^) via the Haber-Weiss and Fenton reactions [90]. Hydrogen peroxide may also react with chloride, a reaction catalysed by myeloperoxidase and yielding hypochlorous acid [111]. 

The reactivity of these various reactive species differs and is influenced by their ability to cross lipid membranes or diffuse in solution. Superoxide is a short-lived, charged molecule that is believed to be only weakly toxic, as it reacts relatively slowly with different biomolecules. It modifies certain small molecules which may result in disruption of oxidative phosphorylation and cellular energy production [78] and does not readily cross phospholipid membranes, acting primarily in proximity to its generation site [112]. 

Hydrogen peroxide is relatively stable under physiological conditions and reacts with a wide range of biologically important compounds. It is readily diffusible within solution and across membranes and can therefore react locally or at a distance from its site of synthesis, depending on numerous factors including the presence of endogenous antioxidants or other ROS [29,78,113].

Hydroxyl radical and peroxynitrite are highly reactive with a wide variety of biomolecules, causing oxidative damage to lipids, proteins, nucleic acids and free nucleotides. Hydroxyl radical is unlikely to travel far from its source due to its high reactivity and peroxynitrite does not freely cross membranes [78]. Hypochlorous acid is a powerful oxidant and potent antibacterial substance that produces a variety of oxidative protein modifications, primarily in areas of inflammation where phagocytes are active. It is regarded by some as a critical killing mechanism for most invading pathogens [114].

## 6. Distribution of NADPH Oxidases in the Brain

Each Nox/Duox enzyme has distinct cell and tissue distribution; most are found abundantly in a primary location or locations and are present at lower levels in a wide variety of tissues [89]. Recent studies on Nox-family enzymes have increasingly shown the importance of deliberate ROS production in various biological events including signal transduction, apoptosis, necrosis, growth and differentiation [78,109]. ROS react indiscriminately with the majority of biomolecules at near-diffusion rate [29]. Therefore, the role of various types of NADPH oxidase-derived ROS in the modulation of cellular functions is dictated by their chemical characteristics, sub-cellular localisation and generation rate. Some cells may co-express several Nox isoforms, for example a constitutively active Nox at the plasma membrane, providing a constant source of ROS at the cell surface and a second Nox isoform in endosomes serving as an agonist-dependent source of oxidant for intracellular signal transduction [89]. 

Important in the brain during ischaemia and reperfusion are those Nox enzymes expressed in neuronal or glial cells, the cerebrovasculature, including smooth muscle cells and endothelium, and in blood-borne immune cells that interact with the vasculature and brain tissue. Subunit expression from all seven Nox/Duox enzymes has been described in brain cells under certain conditions [76,115], however the functionality and specific localisation of all isoforms in the brain has not been reported in depth. Nox2 and Nox4 are reported to be the predominant isoforms expressed in the fore-, mid- and hind-brain, while low levels of Nox1 are also expressed [76]. During ischaemic stroke, excessive Nox-dependent ROS formation, generally associated with the up-regulation of different Nox subtypes, induces dysregulation of the redox control systems and promotes oxidative injury.

### 6.1. Neuronal Nox

NADPH oxidase expression and function has been reported in neurons themselves, as well as in microglia and astrocytes. Initial reports of NADPH oxidase expression in neurons localised p40phox subunit mRNA to selected neuronal populations in the brain [116] and flavocytochrome b558 immunoreactivity to peripheral sensory neurons [117]. Expression of all Nox2 subunits and functional NADPH oxidase activity was reported by Tammarellio *et al.* [118] in sympathetic neurons. Subsequently, the expression and function of Nox2 and related regulatory subunits was confirmed in the central nervous system (CNS), in cultured cortical and hippocampal neurons [119,120,121]. Moreover, *in situ* immunohistochemical analysis of Nox2-related NADPH oxidase subunit expression in normal mouse and rat brain revealed widespread staining in various neuronal populations [122,123]. However, Green *et al.* [124] reported no Nox2 neuronal staining in the normal or ischaemic rat brain, perhaps due to antibody or methodological differences between the studies. Nox4 expression has also been reported in selected neuronal populations in the mouse brain [18], and recently Ha *et al.* [125] reported that the dominant isoforms in primary cortical neurons were Nox4 and Nox2. In contrast, Kahles *et al.* [126] reported Nox1 expression in cultured neurons and suggested that Nox1 had a potentially greater role for ROS production in neurons than Nox2. Nox1 expression was also found to be higher than Nox2 in neuronal PC12 cells, while Nox4 was not detected [127]. These conflicting results could reflect differential Nox regulation in specific neuronal populations or differences in cultured *vs.* *in situ* cells and indicate a need for further investigation. NADPH oxidase-derived ROS play an important role in physiological neuronal function and to date have been linked to various processes including NGF-induced neuronal differentiation and long term potentiation and memory formation [121,128].

### 6.2. Glial Nox

Astrocytes are the predominant type of glial cell in the brain and have been reported to require NADPH oxidase activity for survival [129]. A functional Nox2 NADPH oxidase, which is up-regulated by activation, has been described in cultured cortical astrocytes [13,119,130]. Redox signalling through Nox2 NADPH oxidase plays an essential role in astrocyte function [13], however the expression of Nox2 in astrocytes is lower than that in neutrophils and microglia [13,130]. It has subsequently been shown that cultured rat astrocytes express Nox1, Nox2, Nox4, Duox1 and Duox2 (but not Nox3) [115,126]. Astrocytes play an essential role in maintaining the function of neurons in the brain, including modulation of neural signalling, and also contribute to the formation of the blood brain barrier.

Microglia are the resident macrophages of the CNS. In their activated state they serve a variety of beneficial functions essential for neuronal survival including cellular maintenance and innate immunity [131]. The immunological lineage and inflammatory role of microglia would suggest the presence of a phagocytic Nox2; however conflicting results exist regarding microglial Nox isoform expression. Early studies reported Nox2 expression in both unstimulated and stimulated sheep microglia and, upon activation, superoxide generation in a neutrophil-like manner [132]. These results were confirmed using rat primary cultured microglia, immortalised microglia lines and human foetal microglia cells, which all showed strong expression of Nox2 subunits [13,124]. Nox2 was also reportedly present in activated microglia from a chimpanzee with neuroinflammation [124], and both Nox2 and the regulatory phox subunits were up-regulated upon *in vivo* microglial activation in the rat, generating increased superoxide [133]. Moreover, p47phox, an essential component of the Nox2 NADPH oxidase, was detected in microglial cells from wild type mice, while genetic deficiency in p47phox led to essentially no superoxide production detected in knockout microglial cultures [134]. However, Nox2 mRNA expression by Northern blot was only found in select regions of the normal human brain [124] and the absence of Nox2-related subunit expression has been reported in microglia in normal mouse and rat brain sections [122,123]. In the normal rat CNS, Nox2 expression was only observed in perivascular cells by Green *et al.* [124], hypothesised to be perivascular macrophages. Furthermore, human microglial cell line clone 3 (HMC3), an immortalised cell line exhibiting typical microglial properties, has been reported to express Nox4, rather than Nox2, resulting in constitutive ROS production in these cells [135]. Functional expression of Nox2 and Nox1 (but not Nox3, Nox4, Duox1 or Duox2) has been demonstrated in mouse microglia, with Nox1 playing a role distinct from that of Nox2 in microglial activation, suggested to contribute to microglia neurotoxicity [126,136]. In contrast, low level RT-PCR-detected Nox4 mRNA expression has been reported in cultured rat microglia, in addition to Nox1 and Nox2 [137], and suggested to be involved in excitatory amino acid release via volume-regulated ion channels.

These conflicting results could be due to a complex gene expression profile in microglia, dependent on their activation state; species differences; different gene profiles of *in vivo* cells *vs.* pharmacologically activated cultured cells, and differences in primary cells *vs*. immortalised cell lines. NADPH oxidase has been implicated as both a primary source of microglial-derived extracellular ROS and a mechanism of pro-inflammatory signalling in microglia [131]. Hydrogen peroxide primarily contributes to intracellular cell signalling processes through redox modulation of ion channels, enzymes or transcription factors. In addition, redox modulation of microglial cell morphology, proliferation and function has been attributed to generation of ROS by NADPH oxidase [13,131,138]. Microglia can become overactivated due to direct stimulation by environmental toxins or endogenous proteins, or by neuronal damage and consequent reactive microgliosis [131]. Under pathophysiological conditions, human microglial NADPH oxidase is activated and contributes to neuronal damage in neurodegenerative diseases such as Alzheimer’s and Parkinson’s Diseases [131].

### 6.3. Cerebrovascular Nox

Compared to the work conducted characterising Nox expression activity in peripheral vessels, little has been carried out on the specific expression and regulation of Nox enzymes in the cerebrovasculature. The cerebral circulation is a unique vascular bed with highly specialised physiological mechanisms intended to ensure adequate blood supply to the brain under a wide range of conditions [139]. Nox1, Nox2 and Nox4 expression has been described in cerebral vessels [140], however in comparison to non-cerebral vessels, striking differences in subunit expression and activity have been reported. Under physiological conditions, Nox2 and Nox4 proteins are more highly expressed, and basal and stimulated superoxide generation is greater in intracranial *vs.* systemic arteries [141,142]. Additionally, NADPH-stimulated superoxide production is lower in mice lacking the Nox2 gene than in wild type controls in cerebral, but not systemic arteries, illustrating the differing importance of specific Nox isoform-generated superoxide [141]. In contrast to systemic endothelial cells, significant Nox1 expression has been reported in cerebral endothelial cells [143], though it may not be essential for superoxide generation under basal conditions [144]. Human Nox5 has been shown in low levels in the adult brain [145] and is thought to be an important source of ROS in the vasculature, however because the Nox5 gene is not present in rodents, experimental data is limited [97].

In addition to a unique Nox composition, NADPH oxidase-generated ROS appear to elicit distinct functional responses in cerebral vessels. Under physiological conditions ROS are important signaling molecules for regulation of cerebrovascular function [139]; but in contrast to systemic vessels, a number of ROS have been reported to dilate cerebral arteries, pointing to a functional role for Nox-derived ROS in vasodilator responses. Activation of NADPH oxidase using exogenous NADPH elicits vasodilation in cerebral vessels [146,147], an effect that is attenuated in mice lacking the Nox2 gene [147]. These findings are interesting given that the binding site for NADPH on the NADPH oxidase enzyme is intracellular, and responses are reported after extracellular application of NADPH. There is also substantial evidence that hydrogen peroxide is an important vasoactive molecule in the cerebral circulation and is most commonly reported to elicit dilation in cerebral vessels [139]. Superoxide too, can directly elicit vasodilation in cerebral vessels [140]. 

Despite the apparent differing roles of ROS in cerebral *vs*. systemic vessels, NADPH oxidases play a crucial role in the regulation of cerebral vascular tone and can contribute to cerebrovascular pathologies. In particular, expression of the Nox4 subunit in cerebral vessels appears to be up-regulated in chronic hypertension [146], and cerebral vascular dysfunction in a mouse model of Alzheimer’s disease has been attributed to a Nox2-containing NADPH oxidase [15]. 

### 6.4. Immune Cell Nox

Blood-borne immune cells are activated as part of the inflammatory process and can readily infiltrate the brain upon reperfusion after stroke. Phagocytosis of invading pathogens is the critical physiological function of phagocytes and occurs predominantly within the phagosome. The concentration of superoxide within the phagosome of these cells is very high, however ROS generation may also occur in the extracellular space under a variety of conditions, subjecting surrounding tissues to high ROS levels and resulting in cellular damage [78]. Under these conditions, endogenous antioxidants are crucial in limiting damage. In addition to this role in ROS-mediated destruction of microbes, activation of the phagocyte oxidase is also suggested to have implications for intracellular signalling, related to its function as an electron transporting enzyme. 

The prototypic Nox2 NADPH oxidase is expressed most abundantly in the neutrophil, eosinophil and monocyte/macrophage phagocytic cells. Eosinophils express larger amounts of the phagocytic NADPH oxidase than neutrophils and have a more intense respiratory burst with most stimuli [90]. Monocytes/macrophages express lower levels of the Nox2 and produce less superoxide than neutrophils. When monocytes mature into macrophages their antimicrobial capacity decreases, resulting in diminished oxidative mechanisms [90]. There is little evidence that phagocytes express any Nox isoform other than Nox2, but some studies have demonstrated macrophage Nox1 induction in the setting of atherosclerosis [148,149]. In addition, a recent report suggests that Nox4 is an inducible source of intracellular ROS in human monocytes/macrophages that may play a role in the development and progression of atherosclerosis [150].

## 7. NADPH Oxidase Expression and Activity after Stroke

NADPH oxidase-derived ROS have been increasingly recognised as a primary contributor to oxidative damage following ischaemia and reperfusion injury in the brain. Under normal circumstances, ROS are detoxified by the body’s endogenous antioxidant systems, however when these defence mechanisms are overwhelmed and ROS production exceeds elimination, oxidative cell damage can occur through multiple mechanisms. Therapies involving administration of exogenous antioxidant compounds after stroke have shown benefit primarily in pre-clinical studies and target ROS only after their formation, without addressing the specific processes through which they’re generated. NADPH oxidase expression and activity are increased in the brain after ischaemia-reperfusion [14,16,17,151,152,153] and inhibition of NADPH oxidase has been reported to result in neuroprotection [16,151,154,155], highlighting its potential as a therapeutic target. A notable limitation to pre-clinical studies in the rat and mouse is the absence of Nox5 in their respective genomes and studies investigating the role of Nox5 after ischaemic stroke in different species are lacking.

### 7.1. Nox2

Circulating immune cells known to express Nox2 have been shown to infiltrate the brain after ischaemic stroke [156,157,158,159]. One of the first studies to examine the role of NADPH oxidase in stroke revealed that mice lacking a functional Nox2 component of NADPH oxidase were significantly protected from neuronal injury after cerebral ischaemia [155]. Moreover, generation of chimeric animals by bone marrow transfer indicated that elimination of the Nox2 NADPH oxidase from both circulating phagocytes and endogenous CNS sources was required to confer neuroprotection [155]. This study was recently repeated by Tang *et al.* [160] who reported that Nox2 and resultant ROS originating from circulating cells contributed more to ischaemic damage than that from resident brain cells, emphasising the relevance of targeting the peripheral circulation in stroke treatment. The contrariety between these studies may have arisen due to measures taken by Tang *et al.* [160] to minimize the effect of irradiation by shielding the heads of the mice used. Irradiation can disrupt the blood BBB and injure or activate resident brain cells, however this step was not carried out by Walder *et al.* [155]. Further evidence for the importance of circulating immune cells is provided by the contribution of mature T lymphocytes, which also express a phagocyte-type Nox2-containing NADPH oxidase, to post-stroke superoxide production in the brain [158,161].

It is interesting to note that while the aforementioned studies demonstrate the importance of Nox2-expressing inflammatory cells after stroke, studies investigating outcomes beyond the acute phase of stroke have found that compounds targeting inflammation can be effective with a short survival period, but these initial benefits may be lost over time [162,163]. This phenomenon is not restricted to anti-inflammatory drugs: the NMDA receptor antagonist, MK-801, was found to be neuroprotective at 3 days, but no significant difference in infarct size between treated and placebo rats was evident 28 days after middle cerebral artery occlusion (MCAo) [164]. 

Additional studies have reported that disruption of the Nox2 gene prevented BBB disruption [165] and reduced ROS formation [74], resulting in smaller brain infarcts after stroke. Genetic Nox2 deletion was also shown to provide significant neuroprotection and result in decreased neurological deficits up to 72 h post-ischaemia and reperfusion [166,167]. This was associated with the alleviation of oxidative stress and reduced post-ischaemic neuroinflammation, evidenced by decreased up-regulation of inflammatory mediators and reduced microglial activation [166,167]. Importantly, the level of neuroprotection in Nox2 deficient mice reported by Chen *et al.* [166] decreased at 72 h when compared to 24 h, inviting speculation that Nox2 inhibition may not provide lasting neuroprotection. In contrast to these positive reports, Kleinschnitz *et al.* [16] failed to show a protective effect of genetic Nox2 inhibition at 24 h post-ischaemia and reperfusion. The reasons for this lack of concurrence are unclear but may involve methodological particularities or differences in the genetics of the specific Nox2 mouse colonies used. Nox2 deficient mice were also recently shown to lack protection following permanent ischaemia [168]. This finding suggests that reperfusion is critical for the Nox2 oxidase to play a role in ischaemic brain injury. It provides validation for *in vitro* reports demonstrating the importance of NADPH oxidase for ROS generation during the post-OGD reperfusion phase [25,26,27].

While activation of Nox2-expressing microglia can be protective when resulting in removal of cell debris and killing of pathogens, excessive or chronic activation can kill nearby neurons. Re-oxygenation of microglia after hypoxic/ischaemic insult leads to activation of Nox2 NADPH oxidase and a significant increase in ROS production, resulting in neuronal injury [169,170]. Microglial NADPH oxidase can also regulate the bioavailability of nitric oxide and the production of peroxynitrite [11]. Peroxynitrite generated by glia interacts with both neurons and glia but only induces death in neurons; microglia are essential for this inflammatory-activated glia-induced neuronal death [171].

Expression and activation of neuronal Nox2 has also been implicated in neurotoxicity mediated by oxidative stress [120]. Nox2 in neurons contributed to the ROS and apoptosis induced in a classic programmed cell death model [118], and using an OGD model of brain ischaemia it has been shown that resupply of oxygen and glucose caused an NADPH oxidase-dependent increase in neuronal ROS and cell death, an effect which was absent in p47phox and Nox2 knockout cells [25,27]. There is also pronounced activation of a Nox2 NADPH oxidase in the post-synaptic density after cerebral ischaemia and reperfusion, which may contribute to focal oxidative damage to synaptic function and subsequent development of stroke-induced cerebral injury [153]. In addition to microglial and neuronal NADPH oxidase, astrocytic swelling, which occurs during cerebral ischaemia and reperfusion, triggers p47phox-dependent NADPH oxidase-catalysed ROS production [115]. Astrocytes are relatively resistant to oxidative stress [172] but astroglial ROS may affect neighbouring neurons [173]. In contrast, astrocytes have also been shown to protect against neurotoxicity in some settings [174,175].

Nox2-derived ROS have also been implicated in the progression of stroke using pharmacological inhibition with a number of agents. Atorvastatin (a commonly used cholesterol-lowering medication) was found to suppress NADPH oxidase enzymatic activity by inhibiting Nox2 and p47phox expression, resulting in decreased superoxide levels in the early phase of ischaemia-reperfusion and reduced cerebral infarct compared with non-treated rats [151]. Similarly, betulinic acid administration protected against cerebral ischaemia in mice, in part due to reduction in oxidative stress by down-regulation of Nox2 [152]. Apocynin has been used as a Nox2 NADPH oxidase inhibitor in numerous studies. Apocynin pre-treatment has been shown to reduce infarct size after ischaemia-reperfusion in conjunction with decreased BBB disruption [165], prevention of ischaemia-induced increases in NADPH oxidase activity and superoxide [154,176,177], and a reduction in apoptosis and protein oxidation [153,178]. While it is not an entirely specific Nox inhibitor, apocynin treatment is ineffective at reducing infarct volume in Nox2 knockout mice, an indication that its mechanism of action is strongly mediated through Nox2 inhibition [154]. In contrast to these findings in young adult rodents, apocynin treatment in aged rats is reported to exacerbate stroke injury and diminish functional outcome [179], highlighting the importance of taking into account confounding factors such as advanced age in experimental modeling. In addition, *in vivo* genetic or pharmacological inhibition of Nox2 resulted in altered inflammatory response, increased apoptosis and a trend towards aggravated injury in perinatal mice subjected to hypoxia-ischaemia [180].

### 7.2. Nox1

Comparatively little work has been carried out on the contribution of the Nox1 and Nox4 NADPH oxidases to ischaemic brain damage, mainly due to a scarcity of effective genetic or pharmacologic experimental tools. The effect of Nox1 gene deletion on stroke outcome was first investigated by Jackman *et al.* [144]. Though no effect on total infarct volume was found after 0.5 h MCAo, Nox1 deletion did limit cortical infarct following transient ischaemia and the authors speculated that Nox1 expression may contribute to NADPH oxidase-mediated cerebral vasodilation, offsetting ischaemic damage [144]. Recently, Kahles *et al.* [126] reported that, compared to wild type mice, Nox1 knockout resulted in significant attenuation of lesion volume and neurological deficit, associated with attenuation of BBB disruption, after 1 h of MCAo. Importantly, this protection was not evident using ischaemic periods longer than 1 h [126]. In contrast to these results, Kleinschnitz *et al.* [16] reported no protective effect of Nox1 gene deletion on infarct size after 1 h MCAo. In addition, Nox1 mRNA expression in the brain was reported to be unchanged after 2 h MCAo in ApoE deficient mice [152], and we ourselves were not able to detect relative changes in Nox1 mRNA levels for up to 7 days after stroke in rats [17].

### 7.3. Nox4

Vallet *et al.* [18] were the first to demonstrate the presence of Nox4 expression in neurons. Elevated Nox4 levels were detectable as early as 24 h after stroke, reaching a peak between 7 and 15 days and persisting for up to 30 days, indicating a role for Nox4 in stroke progression and recovery [18]. Subsequently, we showed that Nox4 was also elevated immediately after stroke and reperfusion in rats, but only at 6 h after stroke [17]. In an important finding, Kleinschnitz *et al.* [16] reported the effect of Nox4 gene deletion on outcome after ischaemic stroke. Compared to wild type mice, Nox4 knockouts exhibited significant long-term protection from cerebral ischaemia, independent of age, gender and reperfusion, which was attributed to a reduction in oxidative stress, neuronal apoptosis and BBB leakage [16]. In addition, it was demonstrated that Nox4 immunoreactivity was stronger in brain samples from stroke patients, providing support that these processes are also important in human stroke [16]. Subsequently, a recent report has detailed the effect of endothelial cell-specific Nox4 overexpression, demonstrating a significant increase in infarct volume in transgenic mice compared to controls after permanent MCAo [181]. 

These reports establish the significant effect of Nox4 activity on ischaemic stroke outcome in the absence of reperfusion [16,181], which is in contrast to the recent report on genetic Nox2 inhibition [168]. The protection afforded during permanent ischaemia was less than that provided during temporary ischaemia in Nox4 deficient mice [16], however these results also appear contradictory to *in vitro* studies showing NADPH oxidase-derived ROS were important primarily during reperfusion after OGD [25,26,27]. This highlights a shortcoming of these *in vitro* studies: OGD and reperfusion are applied homogenously to all cells under examination, unlike the *in vivo* context, where heterogeneous changes in cerebral blood flow occur. For example, cells in the penumbra experience less severe blood flow reductions than cells in the ischaemic core, meaning the extent of the oxygen and glucose deprivation in these regions is reduced and may provide for the generation of ROS though NADPH oxidases. Nox4 has been localised to neurons and cerebral blood vessels in the ischaemic brain which may be important sources of oxidative stress in both permanent and transient ischaemia. Indeed, Nox4 is reported to be a dominant Nox isoform in cultured primary cortical neurons and Nox4-derived hydrogen peroxide can result in neurotoxicity [125]. In contrast, reperfusion plays a role in allowing the infiltration of Nox2-expressing circulating immune cells into the ischaemic region, reported to be an important component of Nox2-mediated injury [155,158,160].

## 8. Opposing Roles of NADPH Oxidase in Stroke

### 8.1. Nox and Cerebrovascular Dysregulation after Stroke

As outlined above, Nox1, Nox2 and Nox4 have been implicated in BBB dysfunction during cerebral ischaemia and reperfusion. As with systemic arteries, in general, low concentrations of ROS function as mediators and modulators of vascular homeostasis and cell signaling, but under conditions of oxidative stress ROS produce complex structural and functional changes in the cerebral vessel wall leading to endothelial dysfunction [140]. In several disease models, augmentation of cerebrovascular Nox subunit expression and activity of NADPH oxidases have been reported, leading to a state of oxidative stress [139,140]. The activity of NADPH oxidase is enhanced in cerebral arteries from the ischaemic penumbra following cerebral ischaemia and reperfusion [14]. Moreover, Nox2-mediated excessive superoxide generation, impaired nitric oxide function and nitrosative stress have been shown to occur in mouse cerebral arteries following cerebral ischaemia and reperfusion [182]. When increased levels of superoxide build up in the vascular wall, the major consequence is an increase in vascular tone through the reaction of superoxide with the NOS-derived vasodilator, nitric oxide, which occurs three times faster than the dismutation of superoxide [139]. Peroxynitrite formed through this reaction can reduce the activity of prostacyclin synthase (which generates the vasodilator prostacyclin) and the mitochondrial form of SOD (MnSOD) and lead to the uncoupling of NOS [140]. Additionally, excess hydrogen peroxide can contribute to impaired vascular function through conversion to hydroxyl radical [140]. These processes have extensive implications for regulation of cerebral perfusion and BBB permeability in stroke.

### 8.2. Nox and Oxygen Sensing in the Ischaemic Brain

Various Nox isoforms are also thought to act as part of the oxygen sensing system in the brain, helping to tailor various adaptive responses according to differences in tissue oxygen availability by mediating different regulatory pathways, including induction of hypoxia inducible factor (HIF)-1 [183,184]. HIFs act as key regulators of hypoxia-induced gene expression and regulate gene expression of enzymes or growth factors inducing angiogenesis, anaerobic glycolysis, cell survival or neural stem cell growth [183]. Induction of HIF-1 leads to the up-regulation of HIF-1 regulated gene products such as vascular endothelial growth factor (VEGF). Additionally, HIF-1 can mediate Nox expression and has been shown to up-regulate Nox4-dependent ROS which in turn stimulates the transactivation of growth factor receptors (e.g., VEGFR) and numerous other transcription factors involved in angiogenesis [185]. Furthermore, a functional HIF binding site has been identified on the Nox2 NADPH oxidase [186] and hypoxia has been shown to up-regulate Nox2 mRNA expression in the brain cortex and stem, an effect not observed in Hif1a-deficient mice [187]. Oxygen sensing is particularly important in the brain due to the extreme sensitivity of brain cells to disturbances in oxygen supply. However sustained activation of HIF may lead to a switch from neuroprotective to cell death responses as there are both anti- and pro-apoptotic features of the HIF system [183]. These processes may be particularly relevant to the penumbral region during ischaemic stroke, where some blood supply is conserved but cells may experience different levels of hypoxia.

### 8.3. NADPH Oxidase and Brain Tissue Regeneration

#### 8.3.1. Angiogenesis

It has now been demonstrated that, in contrast to traditional thinking, the adult brain retains some regenerative capacity. After stroke endogenous repair mechanisms such as neurogenesis, angiogenesis, axonal sprouting, and synaptogenesis are enhanced and may contribute to functional recovery. Angiogenesis is the process of new blood vessel formation from the pre-existing vasculature. It is thought to be a key process for endogenous repair, providing enhanced cerebral perfusion and producing neurotrophic compounds that create a suitable microenvironment for the attraction and integration of endogenous or exogenous stem cells [188]. The cerebral vascular system develops exclusively through angiogenesis and, although proliferation of endothelial cells ceases in the adult brain, angiogenesis may still occur under pathophysiological conditions such as stroke [188,189]. Angiogenesis involves both increased proliferation of endothelial cell precursors and migration of vascular cells into the tissue. ROS including superoxide and hydrogen peroxide function as signalling molecules controlling endothelial function and many aspects of growth factor-mediated responses including angiogenesis [190,191]. NADPH oxidases are the primary source of ROS in the vasculature and there is growing evidence that Nox enzymes are important regulators of angiogenesis, both in normal physiology and in pathologies including ischaemic heart failure, cancer, atherosclerosis, and retinopathies [190]. 

**Figure 3:**
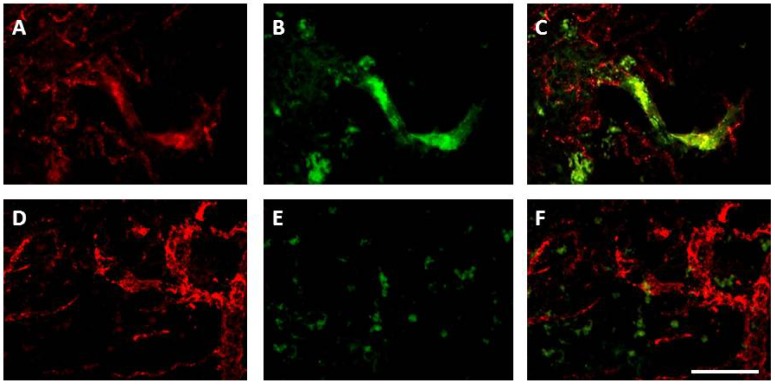
Nox2 immunohistochemistry in the stroke affected cortex is associated with vascular sprouting, in addition to inflammatory cells, 7 days after stroke. Immunofluorescent images of von Willebrand factor labelled blood vessels (red; **A**,**D**) and Nox2 labelled cells (green; **B**,**E**) in the stroke affected cortex 7 days (**A**–**C**) and 28 days (**B**–**E**) after endothelin-1 induced stroke. Merged images (**C**,**E**) reveal angiogenic vessels at 7 days (**C**) are double labelled with Nox2, suggesting a role for Nox2 in vascular sprouting, an effect that is no longer present 28 days after stroke (**E**), once vessels have matured. Scale = 100 µm.

VEGF is a key angiogenic growth factor and stimulates the proliferation and migration of endothelial cells, primarily through VEGF receptor type2 (VEGFR2) [192]. Ischaemia/hypoxia stimulates VEGF and HIF-1 up-regulation through ROS signaling, and binding of VEGF to VEGFR2 stimulates ROS production via activation of NADPH oxidase. In this highly interdependent pathway, the resulting ROS can negatively regulate VEGFR2 [191]. While high concentrations of NADPH oxidase-derived ROS in the acute phase of stroke contribute to oxidative stress and may cause cell death, low levels of ROS, localised intracellularly, function as signaling molecules to mediate angiogenic endothelial cell proliferation and migration in the sub-acute phase of stroke. In the human brain, angiogenesis develops in the penumbra three to four days after stroke [188], and survival time post stroke correlates with the degree of angiogenesis in the damaged tissue [193]. Angiogenesis also occurs in the rodent brain after experimental cerebral ischaemia, initiated in the ischaemic boundary [194]. Using 0.5 h of MCAo in a mouse model of stroke, Hayashi *et al.* [195] reported that proliferating endothelial cells increased as early as one day after stroke and by three days the number of vessels had significantly increased, indicating that angiogenesis begins almost immediately after injury. There is evidence suggesting that Nox1, Nox2, Nox4 and Nox5-derived ROS act as major effectors of pro-angiogenic stimuli (e.g., growth factors, hypoxia) [29]. Nox2-derived ROS have been shown to have an important role in angiogenesis in response to hindlimb ischaemia in the mouse; Nox2 gene deletion results in clear impairment of angiogenesis [196,197]. While angiogenesis is shown to be compromised in Nox2-deficient mice, it is not eliminated. Vallet *et al.* [18] were the first to demonstrate that Nox4, co-localised with new capillaries, was maximally elevated between 7 and 15 days post-stroke in mice, and Nox4 has been shown to positively regulate angiogenic activities *in vitro* [198]. We have reported that Nox4 is also up-regulated in the damaged rat brain in the weeks after stroke, in addition to up-regulation of Nox2 and the most important pro-angiogenic factor, VEGF [199]. Importantly, we have recently shown that Nox2 is co-localised to proliferating blood vessels during angiogenesis 7 days after stroke, an effect no longer detected once new blood vessels have formed (Figure 3, [200]).

While angiogenesis undoubtedly occurs after stroke, its role in the injured brain is somewhat contentious. It has been shown that angiogenesis and neurogenesis are coupled after stroke and that increased angiogenesis leads to enhancement of neurogenesis [201], supporting the theory that angiogenesis is essential for ischaemic brain regeneration. However, others support the “clean up” hypothesis; that the brain uses ischaemia-induced microvessels to provide access for macrophage infiltration and thus facilitate removal of necrotic brain tissue [202], a theory supported by the time-dependent regression of new capillaries and the leaky nature of new vessels [203]. Whatever the role of new vessels after stroke, NADPH oxidase appears to be a key participant in signaling for angiogenesis. Further understanding of angiogenic regulation may allow therapeutic enhancement or manipulation of angiogenesis to improve outcome after stroke.

#### 8.3.2. Neurogenesis

Recent studies suggest that ROS can influence neuronal signaling pathways involved in gene expression, cellular growth, and importantly, differentiation. ROS have been shown to play a role in nerve growth factor-induced terminal differentiation of PC12 cells [128] and depletion of ROS in rat clonal cortical culture shifts the direction of differentiation by increasing the ratio of small to large neurons [204]. Studies have also shown that ROS can influence neurogenesis. High levels of superoxide have been shown to modulate neuronal plasticity in the hippocampus [205], a region of the brain that relies on neural stem cell turnover of neural tissue to facilitate memory and learning [206]. A role for NADPH oxidase in brain neurogenesis has been suggested within the hippocampus since mice and humans that lack functional Nox2 exhibit deficits in learning and memory [207,208]. Most recently it has been reported that within adult hippocampal stem/progenitor cells, hydrogen peroxide derived from Nox2 is essential for normal growth and proliferation *in vitro*. Treatment with hydrogen peroxide triggered a marked increase in phosphorylation of the major growth signaling pathway, Akt, an effect that was abrogated with RNAi knockdown of Nox2 [209]. Furthermore, Nox2 deficient mice show a reduction in the number of newborn hippocampal neurons, supporting a role for NADPH oxidase in neurogenesis *in vivo* [209].

## 9. Inhibitors of NADPH Oxidase

The use of small interfering ribonucleic acids (siRNAs) to specifically inhibit NADPH oxidases are in development, however the specificity and *in vivo* efficacy of many of these siRNAs remains to be confirmed [210,211]. A further complication of this approach is the scarcity of specific antibodies for some Nox isoforms, including Nox4, and reports that Nox4 mRNA levels may not accurately reflect Nox4 protein abundance and activity [125,212]. For these reasons, pharmacological inhibition of NADPH oxidases is a more promising treatment approach for numerous pathologies, including cerebral ischaemia. Studies performed on various experimental animal models, as well as *in vitro*, have demonstrated that pharmacological inhibition of Nox complexes might be a more effective strategy to limiting oxidative damage than scavenging ROS with antioxidant supplementation [78]. However to date, there have been few specific, effective Nox inhibitors available. 

### 9.1. Apocynin

Apocynin (4-hydroxy-3-methoxyacetophenone) inhibits NADPH oxidase activity by interfering with the membrane translocation of p47phox and p67phox and has been used extensively as a Nox inhibitor in stroke studies. It is believed that the inhibitory action of apocynin is not entirely specific to Nox, with some of its inhibitory activity involving myeloperoxidase (MPO) [213,214]. Indeed studies have now shown that MPO together with hydrogen peroxide facilitate apocynin dimerisation and it is these dimers that prevent Nox enzyme assembly upon activation. Despite this, cells that do not contain MPO are still responsive to apocynin, leading to the speculation that it acts as a free radical scavenger [215] rather than a specific Nox inhibitor. This is thought to be true for vascular Nox, however neutrophils have since been shown to secrete MPO *in vivo*, which is then taken up by endothelial cells to convert apocynin into active dimers [216]. 

Retrospective to a specific model of action, the use of apocynin after stroke has been reported to reduce brain injury [154,160,167,176,177]. We too have recently shown that treatment with a high dose of apocynin reduces cortical damage after endothelin-1-induced stroke and reperfusion in rats with a 3 day recovery, but we showed no effect of apocynin treatment on striatal infarcts, as previously reported [217]. This lack of effect was correlated with increased neuronal ROS generation within the penumbra of apocynin-treated rats and we proposed that treatment with apocynin merely delayed the progression of injury over time [217]. Indeed previous reports showing protective actions of apocynin within the striatum measured infarcts only up to 24 h post-reperfusion, at times when the infarct may not have fully matured in the models used for assessment [154,160,167,176,177]. It has been shown previously that ischaemic/reperfusion injury can take at least 3 days post-stroke to reach a maximum in rats with middle cerebral artery occlusion [218]. Furthermore, it is worth noting that investigations using Nox2 deficient mice have not included recovery times beyond 72 h after stroke and long term protection and functional improvement afforded by targeting Nox is yet to be established. This highlights an important need to examine the effects of all potential neuroprotective agents over extended recovery times to ensure that they do not merely delay ischaemic damage, rather than prevent it.

Apocynin treatment has been shown to reduce ROS generation in activated microglia/macrophages after stroke [213,219], and we recently presented a novel finding that apocynin treatment reduces Nox2 expression after stroke without affecting inflammatory cell numbers [217]. Collectively these results suggest that apocynin reduces the inflammatory response, not through reduction of inflammatory cell activation, but potentially through specific effects on Nox2 expression and resulting ROS generation from these cells. To this effect we also reported for the first time differential responses to cell specific ROS generation *in vivo* following treatment with apocynin, in particular an unexpected increase in neuronal ROS 3 days after stroke, compared to vehicle controls [217]. This increase in ROS release from neurons of apocynin treated rats was associated with changes in mitochondrial respiration. The detection of mitochondrial induced oxidative stress within neurons from apocynin treated rats after stroke further supports our hypothesis of an expanding infarct that was delayed due to Nox2 attenuation in inflammatory cells, but later expanded due to changes within neuronal mitochondrial ROS generation. These outcomes again support the idea that several important injury mechanisms overlap and that targeting just one will not result in prevention of injury over time.

Targeting NADPH oxidase with apocynin after stroke is unlikely to result in successful clinical translation. In the setting of experimental stroke, pre-treatment with apocynin is required to show beneficial effects and despite its widespread use, the mechanisms of action and toxicity of apocynin are still poorly understood [154,179]. Furthermore, apocynin has also been shown to affect processes outside of NADPH oxidase activity including arachidonic acid metabolism, nitric oxide synthesis and the cyclooxygenase pathway, and additionally has been shown to act as an antioxidant, rather than a Nox inhibitor in the vasculature [215,220]. Whilst many of these actions may still be beneficial to achieving neuroprotection, unknown systemic toxicity due to the non-specific effects of apocynin, outside its actions on NADPH oxidase, means that it is unlikely to be developed for clinical trial in the near future. Despite this apocynin is indeed a useful tool in which to base future drug development aimed at targeting specific NADPH oxidases. 

### 9.2. DPI

DPI (diphenyleneiodonium) inhibits flavoproteins, affecting the electron-transport capability of NADPH oxidase. Despite being used as a Nox inhibitor in numerous studies, DPI inhibits not only all of the NADPH oxidases, but also other flavoprotein-containing enzymes such as xanthine oxidase, cytochrome P450 reductase, inducible NOS and endothelial NOS [220]. DPI may also stimulate, rather than inhibit ROS generation in certain settings [220]. Limited data exits to show the effects of DPI *in vivo* after stroke, although in combination with dimethylsulfoxide, it is reported to reduce infarct size and BBB disruption following stroke and reperfusion in rats with a 48 h recovery [221].

### 9.3. gp91ds-tat

Peptide inhibitors are a more selective approach to Nox inhibition [140]. The peptide inhibitor gp91ds-tat was designed specifically to affect the interaction of Nox2 with p47phox, inhibiting enzyme assembly and activation [222] and as such presents one of the most specific Nox inhibitors. Based on sequence homology, gp91ds-tat may also affect Nox1 assembly but is unlikely to affect Nox4 [140,222]. However gp91ds-tat has low efficacy in whole cells and all peptide inhibitors, although specific, are not easy to deliver to target tissues as they are not orally bioavailable, presenting a significant barrier to their widespread use [78,220]. Although untested in stroke, gp91ds-tat has been shown to reverse cerebrovascular impairments in aged mice [223].

### 9.4. VAS2870

Many Nox inhibitors developed to date have focused on the effects of attenuating the activity of Nox2 after stroke. Limitations in the available antibodies to specifically label the Nox4 isoform, or prevent its activity, mean that the search for a reliable tool with which to characterise the role of Nox4 in stroke injury is underway. A promising development in this field is outlined in a recent patent review by Kim *et al.* [210] where several new small molecule NADPH oxidase inhibitors showed pre-clinical potency and specificity for individual Nox enzymes. In particular, the triazolopyrimidine derivative, VAS2870, was recently shown by Kleinschnitz *et al.* [16] to have a similarly protective effect as Nox4 knockout in a mouse model of MCAo, where apocynin afforded no protection. Indeed these authors showed significant evidence to suggest that although VAS2870 is non-selective for the Nox isoforms, it was effective in attenuating a Nox4 driven progression of injury in the early hours of stroke. Additionally, a new compound, S17834, has been suggested to inhibit only the vascular Nox but limited information regarding this compound is available, presumably due to commercial sensitivities. S17834 has been shown to inhibit NADPH oxidase activity and decrease apoptotic signalling while not displaying any significant superoxide scavenging properties [224,225]. Hence although these molecules represent the most promising NADPH oxidase inhibitors to date, further safety and efficacy data are needed before such compounds can progress to clinical use [210].

## 10. Future Perspectives

The majority of studies implicate NADPH oxidases as a contributing factor to the pathogenesis of ischaemic stroke. The aforementioned results have led to the promotion of NADPH oxidase as a promising therapeutic target to protect against brain damage [16,129,171,226], if not at least delay its progression [217]. While recently patented Nox inhibitors have shown promising results, caution is needed: disparate results exist between some studies and recent evidence suggests a complex role for ROS in a plethora of cellular functions. Work in systemic vasculature strongly argues for a change in therapeutic strategies to target only deleterious sources of ROS, so as not to affect those that are required for normal vascular function [97]. This is especially important in the tightly-regulated cerebrovasculature where systemic inhibition of Nox may inadvertently compromise cerebral blood flow [142]. In addition, ROS have classically been thought of as pro-inflammatory, however NADPH oxidase activation may also have certain anti-inflammatory or inflammation-regulating effects [227]. Some inhibitors appear to inhibit both vascular and immune cell Nox isoforms and thus if given systemically for extended periods would be expected to compromise immune function [139]. Furthermore, NADPH oxidases may contribute to crucial oxygen sensing and repair processes at particular stages of infarct evolution. Blocking Nox activity indiscriminately may have detrimental effects on cell physiology due to the important roles the Nox enzymes play in the normal physiology of neurons, glia and the cerebrovasculature, and additionally, may affect endogenous post-stroke repair and regeneration processes such as angiogenesis and neurogenesis. Elucidation of the temporal regulation of different Nox isoforms and the relative contribution of neuronal, glial and vascular NADPH oxidases in the pathophysiology of stroke is therefore crucial to ascertain which Nox isoforms need to be targeted at particular time-points to obtain optimum benefit in the acute and sub-acute phases of stroke. To this extent, future studies investigating NADPH oxidase inhibition after stroke need to incorporate longer recovery periods, not only to determine if protection is sustained, but also to investigate what effects this might have on brain repair. 

## 11. Conclusions

A plethora of highly complex mechanisms lead to infarct maturation after ischaemic stroke. While there have been numerous setbacks in the field of ischaemic stroke research, efforts to improve on current treatments and find novel treatment options remain ongoing, with a myriad of therapeutic targets currently under investigation. The role of NADPH oxidase in both the early and the late phase of ischaemia, with or without reperfusion, is yet to be explored with potent, specific inhibitors, but such studies may well offer opportunities for therapeutic intervention in the progression of brain damage following stroke, or in the regeneration phase after stroke. An outstanding feature of all Nox inhibitor studies to date is their lack of inclusion of long term recovery, or extensive investigations into the effects on brain repair mechanisms. These factors are paramount to the clinical development of specific NADPH oxidase inhibitors for ischaemic stroke, as sufferers often survive many years and undergo endogenous repair and reorganisation processes that may be affected by the administration of therapeutics. Thus these elements need to be incorporated into animal models of stroke for successful clinical translation. 
